# Supplementary material for: Pre- and Perioperative Inflammatory Biomarkers in Older Patients Resected for Localized Colorectal Cancer: Associations with Complications and Prognosis
Source: Cancers (Basel). 2021 Dec 29;14(1):161. doi: 10.3390/cancers14010161 (PMC8750535; doi:10.3390/cancers14010161)
Supplement: Supplementary file 1 [file cancers-14-00161-s001.zip › 20211024 REBECCA Cancers supplementary tables S1-3.pdf]

cancers

MDPI

Supplementary material for

Pre- and Perioperative Inflammatory Biomarkers in Older Patients Resected for Localized Colorectal Cancer: Association with Complications and Prognosis

Troels G. Dolin et al.

Supplementary Table S1. Distribution of patients according to age, stage and adjuvant chemotherapy

	<70 years		≥70 years	
	**ACT no, n (%)**	**ACT yes, n (%)**	**ACT no, n (%)**	**ACT yes, n (%)**
Stage I	60 (100)	0 (0)	61 (100)	(0)
Stage II	41 (71)	17 (29)	75 (84)	14 (16)
Stage III	7 (10)	66 (90)	22 (37)	38 (63)

Abbreviations: ACT, Adjuvant chemotherapy.

Supplementary Table S2. Univariate analysis of dichotomized preoperative biomarkers association with major complications, disease recurrence and overall survival

	**Major complications**			**Recurrence**			**Overall Survival**		
	**OR**	**CI**	***P*-value**	**HR**	**CI**	***P*-Value**	**HR**	**CI**	***P*-value**
CRP (all)	1.11	0.51–2.40	0.79	1.40	0.68-2.88	0.36	1.93	1.07–3.49	**0.03**
CRP <70 years	0.29	0.04–2.29	0.24	2.29	0.76-6.84	0.14	2.70	0.89–8.20	0.08
CRP ≥70 years	1.70	0.70–4.14	0.25	0.97	0.37-2.54	0.96	1.50	0.74–3.02	0.14
IL-6 (all)	1.88	1.10–3.22	**0.02**	1.40	0.76-2.59	0.28	1.73	1.02–2.94	**0.04**
IL-6 <70 years	0.85	0.24–3.08	0.81	1.24	0.36-4.24	0.73	1.50	0.43–5.18	0.52
IL-6 ≥ 70 years	2.29	1.09–4.83	**0.002**	1.31	0.63-2.73	0.48	1.40	0.77–2.55	0.27
YKL-40 (all)	1.62	0.94–2.79	0.08	1.09	0.57-2.08	0.80	1.62	0.96–2.75	0.07
YKL-40 <70 years	0.43	0.12–1.50	0.19	0.95	0.32-2.84	0.92	2.51	0.97–6.47	0.06
YKL-40 ≥70 years	3.37	1.57–7.21	**0.002**	1.15	0.52-2.58	0.73	1.29	0.68–2.45	0.44

Biomarkers dichotomized for CRP > 10 mg/L, for IL-6 > 95th percentile of healthy blood donors and YKL-40 > age-corrected 95th percentile. Abbreviations: CI, Confidence Interval; CRP, C-Reactive Protein; HR, Hazard Ratio; IL-6, Interleukin-6.

Supplementary Table S3. Association between different comorbidity scores and performance status and major complications in a multivariate model with YKL-40, age and sex

	**OR**	**CI**	***P*-value**
ASA			0.03
ASA score I vs II	0.29	0.11–0.76	
ASA score II vs III	0.70	0.33–1.64	
CCI			0.007
CCI score 1–2 vs 0	2.58	1.42–4.69	
CCI score ≥ 3 vs 0	1.12	0.23-5.56	
Performance status			0.02
Performance status ≥1 vs 0	2.01	1.11–3.95	

Abbreviations: OR, Odds Ratio; CI, Confidence Interval, ASA, American Society of Anesthesiologists Physical Status Classification System; CCI, Charlson Comorbidity Index
