# Supplementary material for: Pre- and Perioperative Inflammatory Biomarkers in Older Patients Resected for Localized Colorectal Cancer: Associations with Complications and Prognosis
Source: Cancers (Basel). 2021 Dec 29;14(1):161. doi: 10.3390/cancers14010161 (PMC8750535; doi:10.3390/cancers14010161)
Supplement: Supplementary file 1 [file cancers-14-00161-s001.zip › Supplementary Figure S1.pdf]

Supplementary material for

Pre- and Perioperative Inflammatory Biomarkers in Older Patients Resected for Localized Colorectal Cancer: Association with Complications and Prognosis

Troels G. Dolin et al.

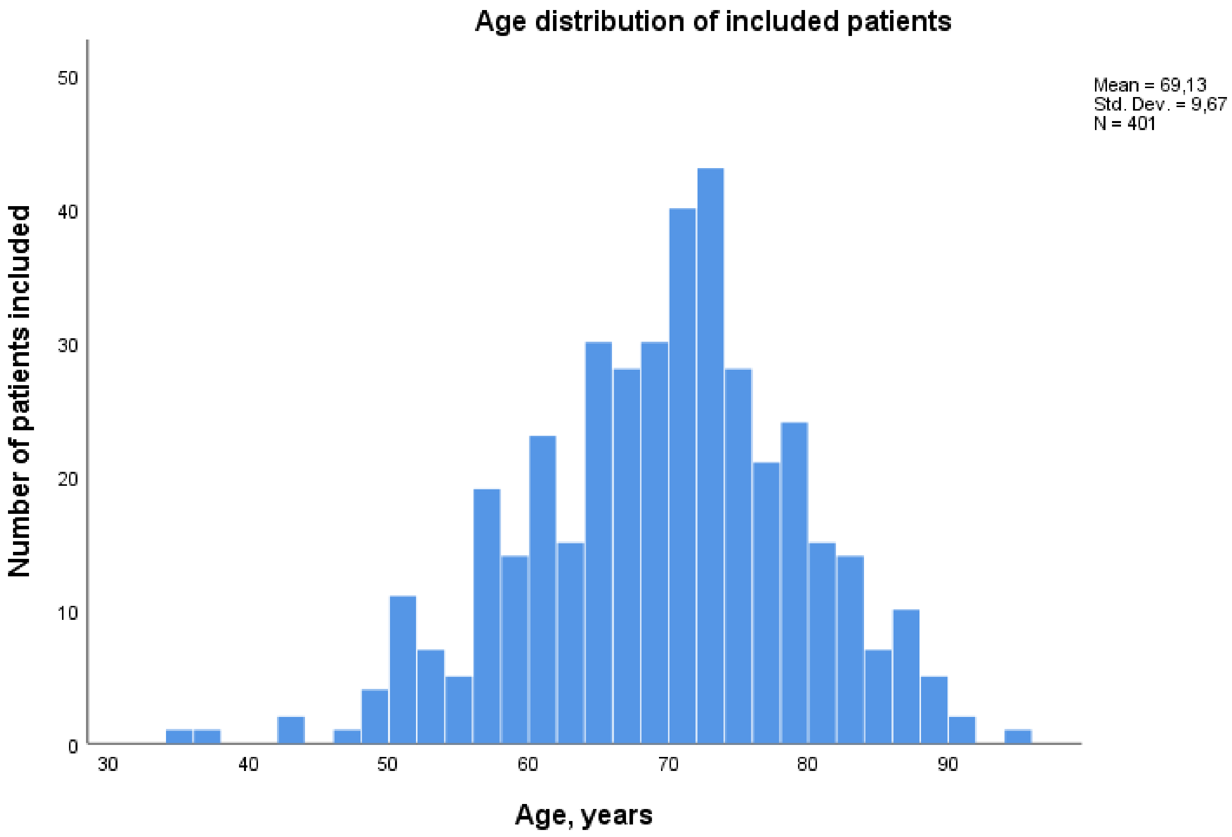

Supplementary Figure S1. Distribution of patients according to age.
