# Supplementary material for: Pre- and Perioperative Inflammatory Biomarkers in Older Patients Resected for Localized Colorectal Cancer: Associations with Complications and Prognosis
Source: Cancers (Basel). 2021 Dec 29;14(1):161. doi: 10.3390/cancers14010161 (PMC8750535; doi:10.3390/cancers14010161)
Supplement: Supplementary file 1 [file cancers-14-00161-s001.zip › Supplementary Figure S2.pdf]

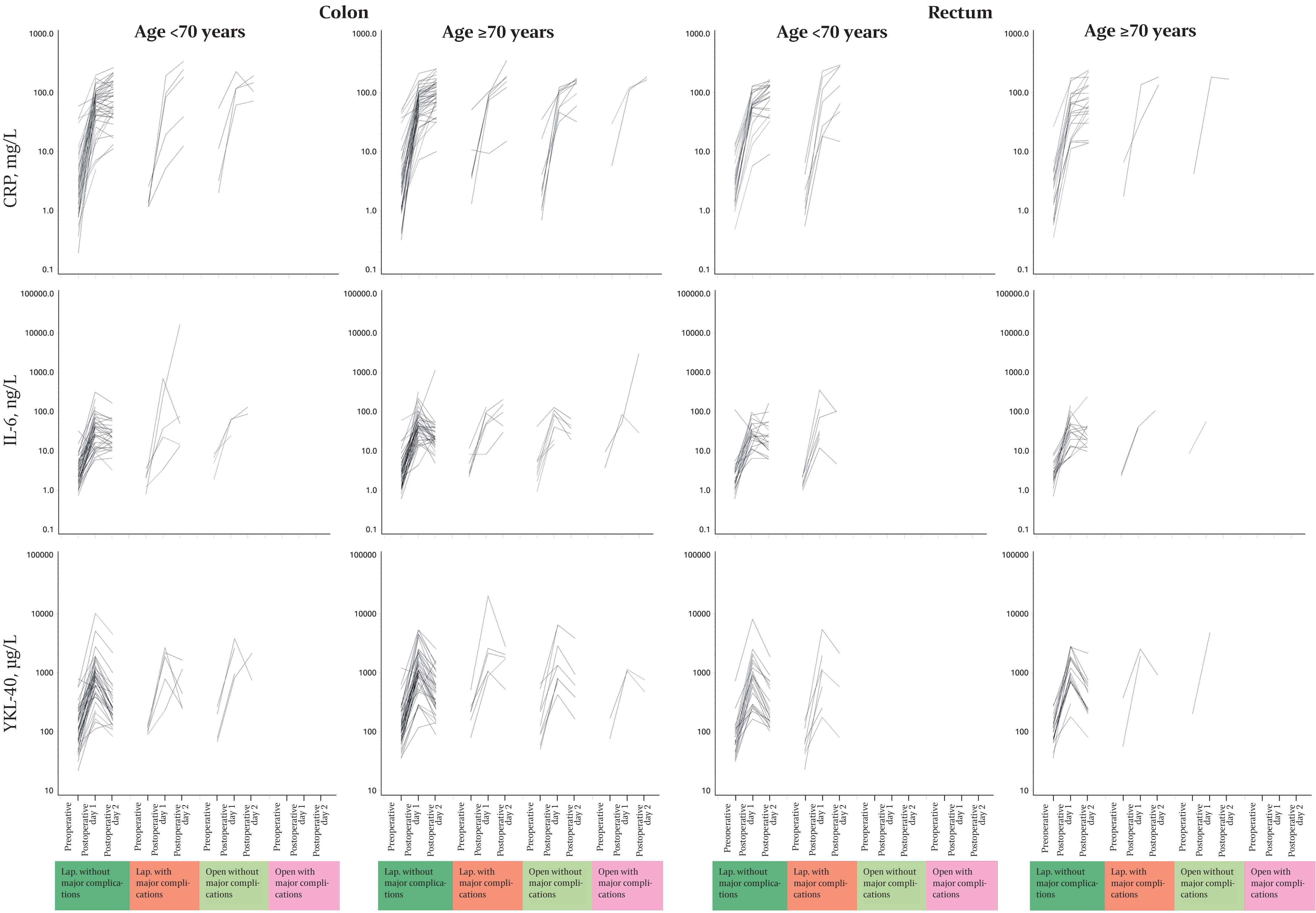

Figure S2. Biomarker changes from preoperative to postoperative stages divided into tumor loca-tion, age, surgical procedure, and complications.
